# Supplementary material for: Intragenic EGFR::EGFR.E1E8 Fusion (EGFRvIII) in 4331 Solid Tumors
Source: Cancers (Basel). 2023 Dec 19;16(1):6. doi: 10.3390/cancers16010006 (PMC10778229; doi:10.3390/cancers16010006)
Supplement: Supplementary file 1 [file cancers-16-00006-s001.zip › cancers-2545478-supplementary.pdf]

Supplementary Materials

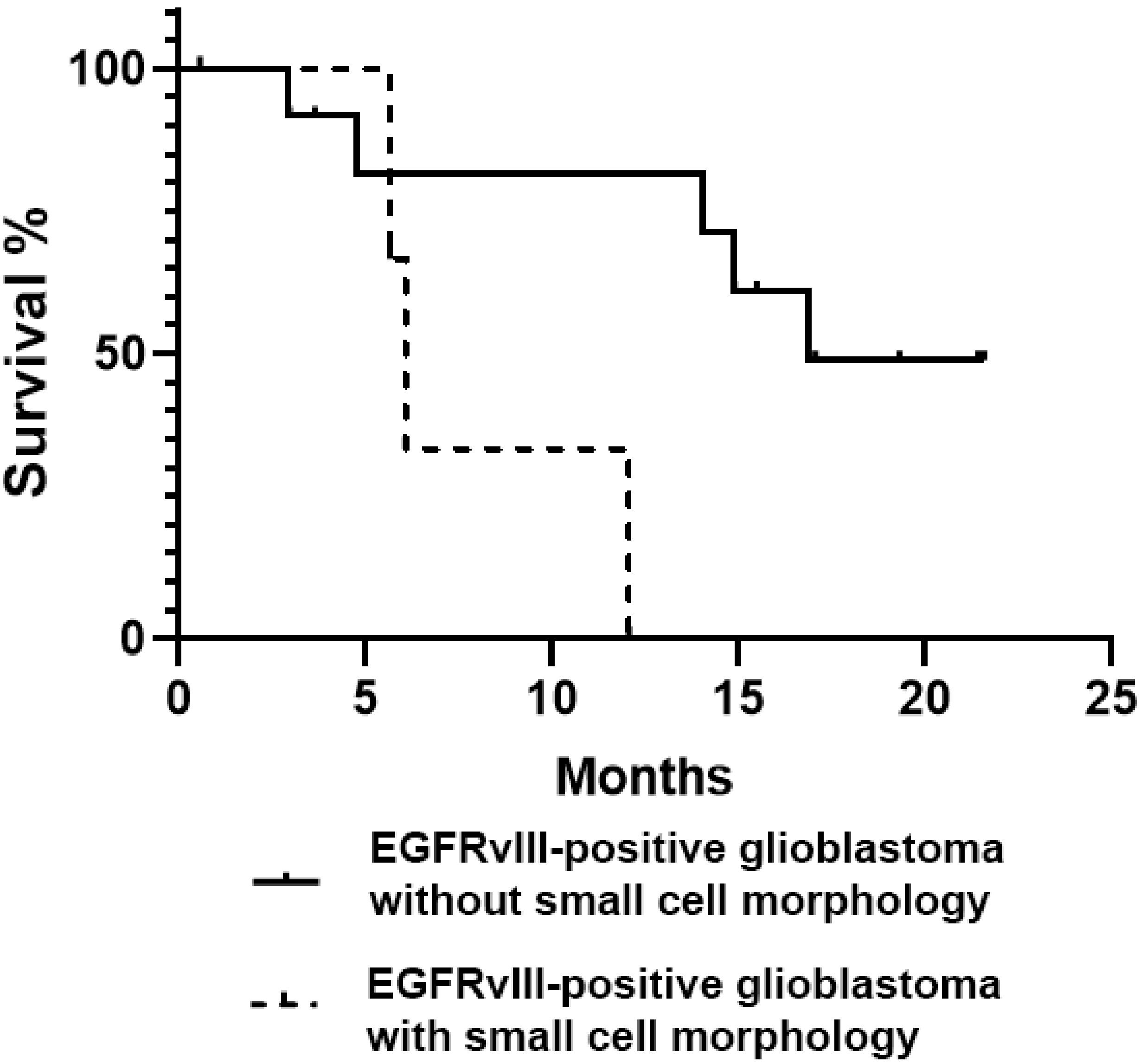

Supplementary Figure S1. Clinical outcome from the presence and absence of small cell morphology in patients with EGFRvIII-positive glioblastoma, IDH-wildtype, CNS WHO grade 4. Kaplan-Meier estimate shows that tumors with reported small cell morphology (dashed line) had a trend for worse overall survival than did tumors without reported small cell morphology (solid line, Log-rank test P = 0.03).

Supplementary Table S1. Clinicopathological characteristics and detected genomic alterations in EGFRvIII-positive glioblastoma.

Case #	Gender	Age	Diagnosis	Amplifications	Fusion	EGFR splice site #	TMB	MSI
N16	F	44	Glioblastoma, IDH-wildtype, CNS WHO grade 4	*EGFR*	EGFR-EGFR.E1E8, EGFR-EGFR.E1E7	4	2 mut/Mb	Stable (MSS)
N15	M	63	Glioblastoma, IDH-wildtype, CNS WHO grade 4	*EGFR*	EGFR-EGFR.E1E8	1	3 mut/Mb	Stable (MSS)
N14	F	76	Glioblastoma, IDH-wildtype, CNS WHO grade 4	*CDK4, EGFR, MDM4, PIK3C2B*	EGFR-EGFR.E1E8	1	4 mut/Mb	Stable (MSS)
N13	M	58	Glioblastoma, IDH-wildtype, CNS WHO grade 4	*None*	EGFR-EGFR.E1E8	1	3 mut/Mb	Stable (MSS)
N12	F	51	Glioblastoma, IDH-wildtype, CNS WHO grade 4	*MDM2*	EGFR-EGFR.E1E8	2	1 mut/Mb	Stable (MSS)
N11	M	52	Glioblastoma, IDH-wildtype, CNS WHO grade 4	*None*	EGFR-EGFR.E1E8	1	1 mut/Mb	Stable (MSS)
N10	M	70	Glioblastoma, IDH-wildtype, CNS WHO grade 4	*EGFR*	EGFR-EGFR.E1E8	1	1 mut/Mb	Stable (MSS)
N09	F	39	Glioblastoma, IDH-wildtype, CNS WHO grade 4	*EGFR*	EGFR-EGFR.E1E8	1	4 mut/Mb	Stable (MSS)
N08	M	55	Glioblastoma, IDH-wildtype, CNS WHO grade 4	*EGFR*	EGFR-EGFR.E1E8	1	4 mut/Mb	Stable (MSS)
N07	M	48	Glioblastoma, IDH-wildtype, CNS WHO grade 4	*EGFR, MDM4, PIK3C2B*	EGFR-EGFR.E1E8	1	1 mut/Mb	Stable (MSS)

N06	M	76	Glioblastoma, IDH-wildtype, CNS WHO grade 4	*CDK4*, *EGFR*, *GLI1*, *MDM2*, *SESN1*	EGFR-EGFR.E1E8	3	2 mut/Mb	Stable (MSS)
N05	M	59	Glioblastoma, IDH-wildtype, CNS WHO grade 4	*EGFR*	EGFR-EGFR.E1E8	2	1 mut/Mb	Stable (MSS)
N04	F	63	Glioblastoma, IDH-wildtype, CNS WHO grade 4	*AKT3*, *EGFR*	EGFR-EGFR.E1E8	2	2 mut/Mb	Stable (MSS)
N03	M	59	Glioblastoma, IDH-wildtype, CNS WHO grade 4	*EGFR*	EGFR-EGFR.E1E8	2	3 mut/Mb	Stable (MSS)
N02	M	65	Glioblastoma, IDH-wildtype, CNS WHO grade 4	*EGFR*	EGFR-EGFR.E1E8, EGFR-EGFR.E1E7	2	5 mut/Mb	Stable (MSS)
N01	F	76	Glioblastoma, IDH-wildtype, CNS WHO grade 4	*EGFR*	EGFR-EGFR.E1E8	3	2 mut/Mb	Stable (MSS)

Supplementary Table S2. Clinicopathological characteristics and detected genomic alterations in sarcomatoid breast tumors.

Case #	Gender	Age	Diagnosis	IHC		Amplification	Fusion	EGFR splice site #	TMB	MSI
				Positive	Negative					
B02	F	15	Sarcomatoid neoplasm	Focal for AE1/AE3, CAM5.2, ERG, p63, S100, and TRPS1; focal and weak for GATA3	CK7, CK903, EMA, CD34, caldesmon, desmin, myogenin, synaptophysin chromogranin, SOX10, ER, PR, HER2	MYC, PTK2, RAD21, RECQL4, RSPO2	EGFR-EGFR.E1E8	1	1 mut/Mb	Stable (MSS)
B01	F	29	Malignant phyllodes tumor	GATA3, TRPS1	Pancytokeratin, AE1/AE3, CK7, CAM5.2, EMA, actin, calponin, CD31, CD34, synapthophysin, chromogranin, S100, SOX10, ER, PR, HER2	EGFR	EGFR-EGFR.E1E8	NA	3 mut/Mb	Stable (MSS)
B03	F	68	Metaplastic carcinoma	Pancytokeratin, AE1/AE3, CK5/6, p63, rare for GATA3	Claudin-4, CD31, CD34, SOX10, ER, PR, HER2	None	None	NA	2 mut/Mb	Stable (MSS)
B04	F	52	Metaplastic carcinoma	CK5/6, p63	ER, PR, HER2	None	None	NA	2 mut/Mb	Stable (MSS)
B05	F	70	Metaplastic carcinoma	Pancytokeratin, GATA3, rare for TRPS1,	CD31, CD34, ERG, ER, PR, HER2	TERT	None	NA	<1 mut/Mb	Stable (MSS)
B06	F	69	Metaplastic carcinoma	Desmin, p63, SMA, vimentin, TRPS1	Pancytokeratin, CD31, ERG, ER, PR, HER2	None	None	NA	4 mut/Mb	Stable (MSS)

NA: Not applicable/not available

Supplementary Table S3: EGFRvIII in histologic subtypes of brain tumors and breast tumors.

	Total Number	EGFRvIII Positive	
		Number	%
**Brain tumors**	**238**	**16**	**7%**
**Astrocytoma, IDH-mutant, CNS WHO grade 2/3**	**37**	**0**	**0%**
Astrocytoma, IDH-mutant, CNS WHO grade 2	26		
Astrocytoma, IDH-mutant, CNS WHO grade 3	11		
**Astrocytoma, IDH-mutant, CNS WHO grade 4**	**26**	**0**	**0%**
**Oligodendroglioma**	**25**	**0**	**0%**
**Diffuse glioma, IDH-wildtype, CNS WHO grade 2/3**	**6**	**0**	**0%**
Diffuse glioma, IDH-wildtype, CNS WHO grade 2	2		
Diffuse glioma, IDH-wildtype, CNS WHO grade 3	4		
**Glioblastoma, IDH-wildtype, CNS WHO grade 4**	**112**	**16**	**14%**
**Astrocytoma, IDH-wildtype, CNS WHO grade 4 (molecular characterized glioblastoma)**	**8**	**0**	**0%**
**Other**	**24**	**0**	**0%**
Diffuse glioma, NEC	1		
Diffuse midline glioma	2		
Ependymoma	8		
Ganglioglioma	1		
Medulloblastoma	2		
Meningioma	5		
Pilocytic astrocytoma	3		
Pleomorphic xanthoastrocytoma	2		
**Breast tumors**	**301**	**1**	**0.3%**
**Ductal**	**253**	**0**	**0%**
**Lobular**	**43**	**0**	**0%**
**Sarcomatoid**	**5**	**1**	**20%**

Supplementary Table S4: Mutations in EGFRvIII-positive glioblastoma, EGFRvIII-positive breast tumors, and metaplastic carcinoma of breast detected by next generation sequencing.

Case #	Gene	DNA	Protein	Location	VAF	Genomic	Type
**N16**	KDM5C	c.3175C>T	p.R1059W	Exon 21	36%	chrX:53224538 G>A	SNV - Missense
	MDM2	c.620T>A	p.V207E	Exon 8	38%	chr12:69222647 T>A	SNV - Missense
	TERT	c.-124C>T		UTR5	49%	chr5:1295228 G>A	SNV
	TRAF2	c.1474A>T	p.I492F	Exon 11	33%	chr9:139820321 A>T	SNV - Missense
**N15**	FOXA1	c.430G>A	p.A144T	Exon 2	8%	chr14:38061559 C>T	SNV - Missense
	JAK3	c.1979C>T	p.A660V	Exon 15	31%	chr19:17945960 G>A	SNV - Missense
	PGR	c.43G>A	p.A15T	Exon 1	40%	chr11:100999759 C>T	SNV - Missense
	PIK3CA	c.3141T>G	p.H1047Q	Exon 21	42%	chr3:178952086 T>G	SNV - Missense
	TBX3	c.438del	p.A147f	Exon 2	37%	chr12:115118903 CT>C	Deletion - Frameshift
	TERT	c.-124C>T		UTR5	44%	chr5:1295228 G>A	SNV
**N14**	EGFR	c.971G>T	p.R324L	Exon 8	17%	chr7:55223604 G>T	SNV - Missense
	EGFR	c.1097G>T	p.S366I	Exon 9	28%	chr7:55224316 G>T	SNV - Missense
	GATA1	c.791C>G	p.T264S	Exon 5	86%	chrX:48651625 C>G	SNV - Missense
	KLF4	c.649C>T	p.Q217*	Exon 3	<5%	chr9:110250026 G>A	SNV - Nonsense
	PGR	c.2450A>T	p.E817V	Exon 6	45%	chr11:100920698 T>A	SNV - Missense
	PIK3CA	c.1258T>C	p.C420R	Exon 8	35%	chr3:178927980 T>C	SNV - Missense
	RFC2	c.787_789delins TTG	p.V263L	Exon 9	15%	chr7:73651743 TAC>CAA	Complex - Missense
	SLIT2	c.1234G>A	p.A412T	Exon 13	7%	chr4:20525486 G>A	SNV - Missense
	SUFU	c.1157+1G>T	p.?	Splice? (Intron 9)	13%	chr10:104375160 G>T	Splice? - Unknown
	TERT	c.-124C>T		UTR5	56%	chr5:1295228 G>A	SNV
	TERT	c.1025G>A	p.R342Q	Exon 2	44%	chr5:1293976 C>T	SNV - Missense
**N13**	KDR	c.2455C>T	p.R819*	Exon 17	44%	chr4:55964358 G>A	SNV - Nonsense
	PIK3R1	c.1651_1656dup AAGCAG	p.K551_Q552dup	Exon 13	37%	chr5:67591063 A>AAGAAGC	Duplication - Duplication
	PIK3R1	c.1658C>T	p.A553V	Exon 13	38%	chr5:67591065 C>T	SNV - Missense
	PLCG1	c.3695G>A	p.R1232Q	Exon 31	44%	chr20:39802813 G>A	SNV - Missense
	RSPO2	c.138T>G	p.C46W	Exon 3	43%	chr8:109001429 A>C	SNV - Missense
	SMC1A	c.1948G>A	p.G650R	Exon 12	43%	chrX:53432287 C>T	SNV - Missense
	TERT	c.-124C>T		UTR5	47%	chr5:1295228 G>A	SNV
	TP53	c.722C>T	p.S241F	Exon 7	44%	chr17:7577559 G>A	SNV - Missense
	TP53BP1	c.5656C>T	p.Q1886*	Exon 27	80%	chr15:43700231 G>A	SNV - Nonsense

**N12**	LRP1B	c.5914C>T	p.H1972Y	Exon 37	35%	chr2:141473651 G>A	SNV - Missense
	PIK3R1	c.1714C>T	p.Q572*	Exon 13	40%	chr5:67591121 C>T	SNV - Nonsense
	PIK3R1	c.218dupA	p.Y73*	Exon 2	31%	chr5:67522721 T>TA	Duplication - Nonsense
**N11**	FGF6	c.189_190delins GC	p.G64R	Exon 1	21%	chr12:4554547 CG>GC	Complex - Missense
	KMT2C	c.1001C>G	p.A334G	Exon 7	<5%	chr7:151970801 G>C	SNV - Missense
	PTEN	c.739_741dup TTA	p.L247dup	Exon 7	38%	chr10:89717716 G>GTTA	Duplication - Duplication
	SUZ12	c.1355G>A	p.W452*	Exon 12	26%	chr17:30320945 G>A	SNV - Nonsense
	TERT	c.-146C>T		UTR5	25%	chr5:1295250 G>A	SNV
**N10**	PTEN	c.1133_1136del	p.R378fs	Exon 9	20%	chr10:89725150 TATAG>T	Deletion - Frameshift
	STAT4	c.2066del	p.G689fs	Exon 22	18%	chr2:191896221 AC>A	Deletion - Frameshift
	TERT	c.-124C>T		UTR5	24%	chr5:1295228 G>A	SNV
**N09**	EGFR	c.787A>C	p.T263P	Exon 7	11%	chr7:55221743 A>C	SNV - Missense
	EGFR	c.685A>T	p.S229C	Exon 6	20%	chr7:55220295 A>T	SNV - Missense
	MAP2K2	c.577C>T	p.R193*	Exon 5	30%	chr19:4101230 G>A	SNV - Nonsense
	MAP3K4	c.4669A>G	p.K1557E	Exon 26	34%	chr6:161536197 A>G	SNV - Missense
	PTEN	c.203A>G	p.Y68C	Exon 3	38%	chr10:89685308 A>G	SNV - Missense
	RB1	c.951_954del	p.S318fs	Exon 10	37%	chr13:48941641 ATCTT>A	Deletion - Frameshift
	SETD2	c.4738delins AGAATATG	p.C1580fs	Exon 6	29%	chr3:47147588 A>CATATTCT	Complex - Frameshift
	TERT	c.-146C>T		UTR5	40%	chr5:1295250 G>A	SNV
	TP53	c.1009C>T	p.R337C	Exon 10	31%	chr17:7574018 G>A	SNV - Missense
	TP53	c.524G>A	p.R175H	Exon 5	29%	chr17:7578406 C>T	SNV - Missense
**N08**	BCORL1	c.1358C>T	p.P453L	Exon 3	35%	chrX:129148106 C>T	SNV - Missense
	CENPE	c.2824C>A	p.Q942K	Exon 23	38%	chr4:104079821 G>T	SNV - Missense
	CIC	c.4284G>C	p.K1428N	Exon 18	14%	chr19:42798413 G>C	SNV - Missense
	COP1	c.1282G>T	p.E428*	Exon 12	10%	chr1:176015456 C>A	SNV - Nonsense
	EGFR	c.866C>T	p.A289V	Exon 7	8%	chr7:55221822 C>T	SNV - Missense
	EGFR	c.986G>A	p.C329Y	Exon 8	19%	chr7:55223619 G>A	SNV - Missense
	TERT	c.-146C>T		UTR5	35%	chr5:1295250 G>A	SNV
**N07**	BLM	c.2725C>G	p.Q909E	Exon 14	38%	chr15:91328213 C>G	SNV - Missense
	PTEN	c.1027-2A>G	p.?	Splice? (Intron 8)	59%	chr10:89725042 A>G	Splice? - Unknown
	TERT	c.-124C>T		UTR5	38%	chr5:1295228 G>A	SNV

	TP63	c.1933A>G	p.T645A	Exon 14	37%	chr3:189612181 A>G	SNV - Missense
**N06**	GATA6	c.1570G>T	p.D524Y	Exon 6	<5%	chr18:19762954 G>T	SNV - Missense
	KIT	c.695C>T	p.T232M	Exon 4	34%	chr4:55565871 C>T	SNV - Missense
	PIK3CA	c.3140A>G	p.H1047R	Exon 21	40%	chr3:178952085 A>G	SNV - Missense
	TERT	c.-146C>T		UTR5	55%	chr5:1295250 G>A	SNV
**N05**	EGFR	c.865G>A	p.A289T	Exon 7	17%	chr7:55221821 G>A	SNV - Missense
	SMC5	c.1180G>A	p.E394K	Exon 9	6%	chr9:72913008 G>A	SNV - Missense
	TERT	c.-124C>T		UTR5	37%	chr5:1295228 G>A	SNV
	TP53	c.1024C>T	p.R342*	Exon 10	75%	chr17:7574003 G>A	SNV - Nonsense
**N04**	EGFR	c.1952T>A	p.V651E	Exon 17	<5%	chr7:55240708 T>A	SNV - Missense
	ERBB3	c.1468C>T	p.R490C	Exon 12	38%	chr12:56487322 C>T	SNV - Missense
	TERT	c.-124C>T		UTR5	36%	chr5:1295228 G>A	SNV
	TOP3A	c.517G>A	p.V173M	Exon 6	39%	chr17:18206020 C>T	SNV - Missense
**N03**	EGFR	c.866C>T	p.A289V	Exon 7	26%	chr7:55221822 C>T	SNV - Missense
	EGFR	c.3056C>T	p.P1019L	Exon 25	<5%	chr7:55268990 C>T	SNV - Missense
	PRKDC	c.11948T>G	p.M3983R	Exon 85	31%	chr8:48690338 A>C	SNV - Missense
	TERT	c.-146C>T		UTR5	46%	chr5:1295250 G>A	SNV
	TP53	c.272G>A	p.W91*	Exon 4	33%	chr17:7579415 C>T	SNV - Nonsense
**N02**	AURKC	c.333G>A	p.?	Splice? (Exon 4)	7%	chr19:57744048 G>A	SNV - Unknown
	EGFR	c.2320G>A	p.V774M	Exon 20	<5%	chr7:55249022 G>A	SNV - Missense
	FLT4	c.3461G>C	p.G1154A	Exon 26	10%	chr5:180039582 C>G	SNV - Missense
	LRP1B	c.10310C>G	p.S3437C	Exon 66	8%	chr2:141200177 G>C	SNV - Missense
	NEGR1	c.37T>C	p.S13P	Exon 1	10%	chr1:72748141 A>G	SNV - Missense
	PIK3CA	c.353G>A	p.?	Splice? (Exon 3)	7%	chr3:178917478 G>A	SNV - Unknown
	ROS1	c.1040C>T	p.A347V	Exon 10	8%	chr6:117715449 G>A	SNV - Missense
	TERT	c.-146C>T		UTR5	14%	chr5:1295250 G>A	SNV
	TMEM127	c.245A>G	p.?	Splice? (Exon 3)	9%	chr2:96920735 T>C	SNV - Unknown
**N01**	CARD11	c.1310G>A	p.R437H	Exon 9	22%	chr7:2976702 C>T	SNV - Missense
	EGFR	c.754C>T	p.R252C	Exon 7	19%	chr7:55221710 C>T	SNV - Missense
**B02**	PIK3CG	c.935A>G	p.D312G	Exon 2	20%	chr7:106508941 A>G	SNV - Missense
	TP53	c.782+1G>A	p.?	Splice? (Intron 7)	86%	chr17:7577498 C>T	Splice? - Unknown

**B03**	EPHA3	c.2231A>G	p.H744R	Exon 13	<5%	chr3:89480394 A>G	SNV - Missense
	NRAS	c.35G>T	p.G12V	Exon 2	46%	chr1:115258747 C>A	SNV - Missense
	PIK3CA	c.3140A>G	p.H1047R	Exon 21	32%	chr3:178952085 A>G	SNV - Missense
	RMI1	c.1121G>A	p.S374N	Exon 3	8%	chr9:86617022 G>A	SNV - Missense
	SGK1	c.714_717del	p.D240fs	Exon 8	34%	chr6:134493400 CAGTA>C	Deletion - Frameshift
	SOS1	c.2330T>A	p.L777H	Exon 14	<5%	chr2:39239327 A>T	SNV - Missense
	TCF3	c.1443_1447 delinsT	p.Y482fs	Exon 16	<5%	chr19:1619113 TGTAG>A	Complex - Frameshift
	TERT	c.-124C>T		UTR5	57%	chr5:1295228 G>A	SNV
**B04**	BAP1	c.67+1G>C	p.?	Splice? (Intron 2)	16%	chr3:52443729 C>G	Splice? - Unknown
	NOTCH2	c.2570A>T	p.Y857F	Exon 16	28%	chr1:120491659 T>A	SNV - Missense
	PIK3R1	c.1966dupT	p.C656fs	Exon 15	20%	chr5:67592150 C>CT	Duplication - Frameshift
	PIK3R1	c.1727_1735 dupCGAGAGACC	p.D578_Q579 insPRD	Exon 13	15%	chr5:67591142 A>ACGAGAGACC	Duplication - Insertion
	TGFBR1	c.238C>T	p.R80*	Exon 2	38%	chr9:101891277 C>T	SNV - Nonsense
	TP53	c.817C>T	p.R273C	Exon 8	6%	chr17:7577121 G>A	SNV - Missense
**B05**	ASXL2	c.649C>T	p.Q217*	Exon 8	22%	chr2:25990578 G>A	SNV - Nonsense
	HRAS	c.182A>T	p.Q61L	Exon 3	24%	chr11:533874 T>A	SNV - Missense
	PAK3	c.546_548dupAGA	p.E182dup	Exon 9	25%	chrX:110406222 G>GGAA	Duplication - Duplication
	PIK3CA	c.3140A>G	p.H1047R	Exon 21	25%	chr3:178952085 A>G	SNV - Missense
	TERT	c.-124C>T		UTR5	95%	chr5:1295228 G>A	SNV
**B06**	FGFR2	c.1942G>A	p.A648T	Exon 14	10%	chr10:123247549 C>T	SNV - Missense
	FLT1	c.841C>T	p.R281*	Exon 7	34%	chr13:29005420 G>A	SNV - Nonsense
	KMT2C	c.1519C>T	p.Q507*	Exon 11	56%	chr7:151949126 G>A	SNV - Nonsense
	NF1	c.4230_4245 delinsG	p.F1410_N1415 delinsL	Exon 32	82%	chr17:29585418 CCTCAGATTTATCAAT>G	Complex - Complex
	PIK3CA	c.1624G>A	p.E542K	Exon 10	42%	chr3:178936082 G>A	SNV - Missense
	PTEN	c.465T>G	p.Y155*	Exon 5	43%	chr10:89692981 T>G	SNV - Nonsense
	RASA1	c.1984_1988del	p.T662fs	Exon 15	83%	chr5:86670706 TAAAAC>T	Deletion - Frameshift
	SLIT2	c.2614G>A	p.D872N	Exon 26	7%	chr4:20555480 G>A	SNV - Missense
	SMAD4	c.1592G>A	p.R531Q	Exon 12	86%	chr18:48604770 G>A	SNV - Missense
	TERT	c.581G>A	p.R194Q	Exon 2	15%	chr5:1294420 C>T	SNV - Missense
	TP53	c.375G>A	p.?	Splice? (Exon 4)	84%	chr17:7579312 C>T	SNV - Unknown
